# Editorial: Racial, ethnic, and other inequalities in healthcare and in pain reporting and management

**DOI:** 10.3389/fpain.2023.1275137

**Published:** 2023-11-20

**Authors:** Shivantika Sharad

**Affiliations:** Department of Applied Psychology, Vivekananda College, University of Delhi, New Delhi, India

**Keywords:** inequality, healthcare, stigma, culture, discrimination, stereotype, health communication

**Editorial on the Research Topic** Racial, ethnic, and other inequalities in healthcare and in pain reporting and management

Inspired by the fight against social discrimination, the research topic “Racial, ethnic and other inequalities in healthcare and in pain reporting and management” is a pertinent issue faced by society, more so in the aftermath of the COVID-19 pandemic that saw huge inequalities in healthcare access across the globe (1). People from underprivileged backgrounds face many predicaments with their health needs being ignored and tremendous inequalities in health access, reporting, and health outcomes (2). Health inequalities due to race (3, 4), ethnicity (5), socioeconomic status, age (6), gender (7), religion, sexual orientation, and gender are widespread in the world.

Thus, this research topic aimed to underscore the pressing issue of health disparities by presenting studies in the area and exploring ways of amelioration. Four papers were submitted under the topic. All four contributing articles were original research papers dealing with different minority groups from three different continents, namely, Europe, Australia, and Asia.

The first paper was a qualitative study dealing with the access to healthcare for the Aboriginal and Torres Strait Islander (ATSI) people in Queensland, Australia (Bernardes et al. 2022). A total of 21 individuals including both patients and hospital liaison officers from the same community ATSI participated in interviews and focus group discussions. The study explored the communication experiences of patients and health officials on pain management. Experiences of stigma, negative stereotypes, and non-disclosure of ATSI identity for fear of discrimination in terms of reduced opportunities for treatment comprised the emotional burden of the patients. The study highlighted the role of acknowledging these historical and cultural factors to establish trust between patients and health professionals and to overcome the difficulties in using standard pain measurement scales with specific ethnic groups. Pain management services need to be patient-centric and adopt a model of care that imbibes greater cultural understanding and respect for diversity to improve its access and effectiveness.

The second paper was a quantitative study on 220 South Korean patients with chronic secondary musculoskeletal pain in rheumatic diseases, exploring biological, socioeconomic, and psychological factors associated with clinical pain intensity and interference among patients (Kim et al. 2023). Sex differences in the role of these factors were also examined. Both pain intensity and interference were positively related to psychological factors, namely, depression and pain catastrophizing. Sex differences were found in the nature of the influence of these psychological factors. Age was associated with pain interference in men, while depressive symptoms were associated with pain interference in women. Women were more directly affected by depressive symptoms than men, regarding pain intensity and interference. The study recommended a sex-specific approach to the biopsychosocial model for understanding and managing pain among Asians. It also endorsed integrating the psychological factors in pain assessment to get a complete picture of pain experience.

Research by Wu et al. (2023) on the unmet healthcare needs, health outcomes, and health inequalities among the Chinese elderly is an important contribution. The study showed how elderly people with additional disabilities, illnesses, and social needs are more vulnerable and have not been receiving the needed healthcare in China. Lack of access to healthcare services and unmet health needs adversely impacted health outcomes, including depression. It was found that elderly people with frailer health were more likely to have unmet needs, which was affected by affordability (economically poor). In addition, the relatively healthy elderly people were most affected by unmet needs due to lack of availability. It is also important to note that people with greater unmet health needs (frailer people) were older, less educated, living in rural areas, and having lower income and lower living standards, and of these, more than 60% were women. On the other hand, healthier elderly people were more educated and were from urban areas. These results pointed out a significant health inequity that exists in China and how healthcare access and benefits need to be equally distributed in rural and urban areas. In addition, more emphasis needs to be given to elderly people who are frailer and poorer.

The last contribution to the topic was qualitative research based on interviews with 39 healthcare professionals (Orzechowski, 2023). The study aimed to understand the experiences and attitudes of healthcare professionals toward the issue of social diversity and disadvantaged group’s access to healthcare in Croatia, Germany, Poland, and Slovenia. Thematic analysis revealed that the healthcare workers acknowledged the impact of socioeconomic factors and minority group membership on accessing healthcare services in all four countries, with varying degrees. The findings emphasized the need for diversity-responsive healthcare in the face of challenges like underfunding healthcare, language barriers, inadequate cultural training or lack of interpersonal competencies for healthcare workers, and lack of institutional support. Although direct forms of systemic exclusion of the minority groups were not reported, individual instances of discrimination (gossip, stereotyping, stigma, and inappropriate communication) based on homophobia and racism were shared. Language barrier at the systemic level, with no provisions for trained medical interpreters, was also cited as a major limitation in the effective treatment of minorities. It was suggested that equality in healthcare access by minority groups could be attained by educating healthcare professionals in diversity awareness and building their diversity competency to efficiently and compassionately deal with patients from diverse backgrounds.

These studies, taken together, direct our attention to the need for integrating diversity awareness, cultural sensitivity, and cultural competencies in healthcare systems at all levels. Efforts are solicited to bridge the gap between the healthcare needs of underprivileged communities and healthcare access and provision all across the world.
